# Cell‐permeable JNK‐inhibitory peptide regulates intestinal barrier function and inflammation to ameliorate necrotizing enterocolitis

**DOI:** 10.1111/jcmm.18534

**Published:** 2024-07-19

**Authors:** Chaozhi Bu, Mengyuan Hu, Yinglin Su, Fuqiang Yuan, Yiting Zhang, Jing Xia, Zhenyu Jia, Le Zhang

**Affiliations:** ^1^ Department of Neonatology Affiliated Children's Hospital of Jiangnan University (Wuxi Children's Hospital) Wuxi China; ^2^ State Key Laboratory of Reproductive Medicine, Research Institute for Reproductive Health and Genetic Diseases, Wuxi Maternity and Child Health Care Hospital Women's Hospital of Jiangnan University, Jiangnan University Wuxi China; ^3^ Department of Neonatology The Affiliated Wuxi Children's Hospital of Nanjing Medical University Wuxi Jiangsu China; ^4^ Department of Gastroenterology and Digestive Diseases The First Affiliated Hospital of Soochow University Suzhou Jiangsu China

**Keywords:** CPJIP, intestinal barrier, JNK, necrotizing enterocolitis, tight junction

## Abstract

Intestinal dysbiosis is believed to play a role in the development of necrotizing enterocolitis (NEC). The efficacy of JNK‐inhibitory peptide (CPJIP) in treating NEC was assessed. Treatment with CPJIP led to a notable reduction in p‐JNK expression in IEC‐6 cells and NEC mice. Following LPS stimulation, the expression of RNA and protein of claudin‐1, claudin‐3, claudin‐4 and occludin was significantly decreased, with this decrease being reversed by CPJIP administration, except for claudin‐3, which remained consistent in NEC mice. Moreover, the expression levels of the inflammatory factors TNF‐α, IL‐1β and IL‐6 were markedly elevated, a phenomenon that was effectively mitigated by the addition of CPJIP in both IEC‐6 cells and NEC mice. CPJIP administration resulted in improved survival rates, ameliorated microscopic intestinal mucosal injury, and increased the total length of the intestines and colon in NEC mice. Additionally, CPJIP treatment led to a reduction in serum concentrations of FD‐4, D‐lactate and DAO. Furthermore, our results revealed that CPJIP effectively inhibited intestinal cell apoptosis and promoted cell proliferation in the intestine. This study represents the first documentation of CPJIP's ability to enhance the expression of tight junction components, suppress inflammatory responses, and rescue intestinal cell fate by inhibiting JNK activation, ultimately mitigating intestinal severity. These findings suggest that CPJIP has the potential to serve as a promising candidate for the treatment of NEC.

## 1 INTRODUCTION

Necrotizing enterocolitis (NEC) is a serious condition that affects premature neonates and is marked by heightened intestinal inflammation, elevated levels of cytokines including interleukin 1α (IL‐1α), IL‐1β, IL‐6 and tumour necrosis factor‐alpha (TNF‐α), and rapid necrosis of the intestinal tissue, leading to mortality in more than one‐third of cases.^1^, ^2^ Multiple factors contribute to the hyperinflammatory intestinal features in NEC, including the immaturity of the intestinal immune system, intestinal ischemia, barrier dysfunction, motility and local circulatory disorders and intestinal dysbiosis.^3^, ^4^ Despite numerous advancements in experimental research on the pathogenesis of NEC, the exact mechanisms are not yet fully elucidated.

The intestinal epithelial cells, in conjunction with the tight junction (TJ), constitute the inherent intestinal epithelial barrier, which functions as a physical and functional barrier against the trans‐epithelial permeation of luminal substances.^5^ Prior research has suggested that the build‐up of pro‐inflammatory cytokines such as TNF‐α and IL‐1β can trigger antigenic penetration, resulting in an increase in intestinal TJ permeability.^6^, ^7^, ^8^ The established literature indicates that the compromised integrity of the intestinal TJ barrier, coupled with heightened inflammation, facilitates the transcellular passage of luminal antigens, thereby exacerbating intestinal damage and inflammatory responses in conditions characterized by increased intestinal permeability, such as inflammatory bowel disease and NEC.^9^, ^10^, ^11^ Studies have shown that maintaining the integrity of the intestinal TJ barrier is effective in mitigating intestinal inflammation and enhancing intestinal permeability in cases of NEC.^12^, ^13^ Cytoplasmic and transmembrane TJ proteins, such as occludin, ZO, claudin family and junctional adhesion molecules, have been demonstrated to function as sealing elements and participate in signal transduction, regulation of cellular motility and intracellular trafficking.^14^, ^15^

The activation of the c‐Jun N‐terminal kinase (JNK) family by cytokine stimulation and physiological stressors is implicated in the disassembly of TJ proteins and the regulation of intestinal permeability, which are associated with the pathogenesis of NEC.^16^, ^17^ Nevertheless, further investigation is required to elucidate the precise mechanism by which TJ proteins interact with the JNK pathway to contribute to the development of NEC.

Various prevention methods, including the administration of probiotics, proteins, and amino acids such as antimicrobial peptides, whey‐derived antioxidative peptide, intestinal alkaline phosphatase, casein‐derived peptide and lactoferrin, have been utilized as feeding interventions to mitigate intestinal inflammation‐induced tissue injury, promote intestinal epithelial cell migration, and maintain epithelial barrier integrity in NEC.^18^, ^19^, ^20^ In this study, we utilized a cell‐permeable JNK‐inhibitory peptide (CPJIP) known for its ability to regulate glucose metabolism and improve diabetes, to investigate its effectiveness in regulating experimental NEC both in vitro and in vivo. This study aims to assess the efficacy of this pharmaceutical approach and elucidate the underlying pathological mechanisms of NEC, with the ultimate goal of identifying a novel therapeutic candidate for this condition.

## 2 MATERIALS AND METHODS

### 2.1 Peptide synthesis

Peptide CPJIP was synthesized by Shanghai Science Peptide Co. Ltd. The purity of peptides was determined by high‐performance liquid chromatography (HPLC) and exceeded 95%. The peptides were dissolved in endotoxin‐free water and stored at −80°C.

### 2.2 Cell culture

The rat small intestinal crypt cell line (IEC‐6) was obtained from the typical culture preservation commission cell bank of the Chinese Academy of Sciences in China and cultured in Dulbecco's Modified Eagle's Medium (Gibco, USA), supplemented with fetal bovine serum (FBS) (10%, Gibco, USA) and penicillin–streptomycin (1%, Gibco, USA). Cells were maintained in a humidified incubator at 37°C with 5% CO_2_. Lipopolysaccharides (LPS, 100 μg/mL, Sigma‐Aldrich, USA) was used for establishment of in vitro NEC model. CPJIP was pretreated at the concentrations of 100 μM for 3 h before LPS stimulation.

### 2.3 Evaluation of cellular uptake and in vivo distribution

CPJIP was labelled with fluorescein isothiocyanate (FITC). For in vitro assessment, CPJIP (4 μM) was added into the medium IEC‐6 cells for 1 h. Then IEC‐6 cells were washed three times with 1 × PBS and fixed in 4% paraformaldehyde (Solarbio, China), and stained with DAPI (Solarbio, USA). Fluorescence images were obtained using a microscope (Nikon, Japan). In vivo biodistribution of CPJIP in intestinal tract was carried out using small animal live imaging system (IVIS Spectrum, Perkin‐Elmer, USA). Briefly, two group of mice were given orally with or without a single dose (10 mg/kg) of FITC‐labelled CPJIP for 1 h. After that, the fluorescence images of intestinal tissues dissected and separated from mice were captured.

### 2.4 Establishment of neonatal NEC mouse model and evaluation of clinical sickness score

Experimental NEC was induced in 7‐day‐old mice of either gender, which were randomly divided into four groups: Control, NEC, CPJIP and NEC + CPJIP. The mice in the control and CPJIP groups were allowed to nurse their mothers freely, while CPJIP group mice were fed with additional CPJIP. For NEC group, mice were daily intragastric administrated with hypertonic milk (Wyeth Stage 1:Abbott Birth Puppy Formula 2:1, at 50 μL/mg body weight and 200 μg of CPJIP was added per ml of hypertonic milk in NEC + CPJIP group). After the pups in the experimental group and the intervention group were fed the hypertonic milk, they were subjected to hypoxia (5% O_2_–95% N_2_) for 10 min in a hypoxia chamber twice daily for 4 days. The severity of disease was determined on histologic sections of the small intestines stained with haematoxylin and eosin. Furthermore, the clinical disease score was assessed daily by an impartial observer based on the mice natural physical activity, body colour, response to tactile stimulus, and overall appearance during feeding times. Each category was assigned a score ranging from 0 to 3, with a total clinical disease score ranging from 0 (best) to 12 (worst). The animal study was reviewed and approved by the Institutional Animal Care and Use Committee at Affiliated Wuxi Children's Hospital of Jiangnan University (No. WXCH2021‐09‐012).

### 2.5 Histology and scoring

The intestine was dissected from the body of each group of mice, and small segments of colon were obtained and fixed with 4% paraformaldehyde for 24 h at room temperature. Then, collected samples were dehydrated and embedded in paraffin and were cut into 4 μm of slices, serially stained with haematoxylin and eosin (HE, Solarbio, China). The histopathological examination of haematoxylin and eosin stained sections revealed changes that were scored on a scale ranging from 0 to 4: (0) Normal histology; (1) Focal mild changes limited to intestinal villi; Hydropic degeneration, minimal detachment of superfcial epithelial cells from the lamina propria and/or detachment from the submucosa; (2) Villus epithelial necrosis and thinning of the intestinal wall; (3) Necrosis extending to the submucosa; (4) Transmural necrosis.

### 2.6 Immunohistochemistry

Paraffin sections used for immunohistochemistry were deparaffinized and rehydrated with xylene and ethanol, respectively. Antigen retrieval was performed for sections using citric acid antigen retrieval buffer. Then, the sections were incubated in 3% hydrogen peroxide at room temperature in the dark for 25 min and washed with PBS. Sections were blocked in 1% normal goat serum and incubated overnight at 4°C with antibodies specific for claudin‐1, claudin‐3, claudin‐4 and occludin (ThermoFisher, USA), followed by incubation with horseradish peroxidase (HRP)‐coupled secondary antibodies. A chromogenic reaction was performed with DAB colour developing solution (Solarbio, China) and staining images were visualized using an optical microscope (Nikon Eclipse Ni, Japan). Mean integral optical density was obtained using Image J software and statically analysed.

### 2.7 TUNEL (terminal deoxyribonucleotide transferase‐mediated nick‐end labeling) assay

The TUNEL assay was performed on mouse intestine sections using a TUNEL BrightGreen Apoptosis Detection Kit (Vazyme, China) according to the manufacturer's protocols. Intestinal colon tissues were fixed in 4% formaldehyde, dehydrated by a graded ethanol series, embedded in paraffin, and sliced into sections. Then sections were dewaxed, rehydrated, permeabilized with proteinase K. After washing with PBS, the slides were stained with 2 μg/mL DAPI for 5 min in the dark. Sections were observed with a confocal laser scanning microscope (Ti2, Nikon).

### 2.8 EdU staining assays

The proliferation of intestinal cells in four individual groups was evaluated using an EdU cell proliferation detection kit (Beyotime, China) according to the manufacturer's protocol. Mean integral optical density was obtained using Image J software.

### 2.9 Enzyme‐Linked Immunosorbent Assay (ELISA)

The levels of the inflammatory cytokines IL‐1β, IL‐6 and TNF‐α were measured in the supernatant of culture medium in IEC‐6 cells with rat IL‐1β, IL‐6 and TNF‐α ELISA kits (MEIMIAN, China), respectively, according to the manufacturer's instructions. In addition, serum was harvested from mice 4 h after LPS treatment, and the levels of diamine oxidase (DAO) and D‐lactate were examined using ELISA kits (Bioswamp) following the manufacturer's instructions.

### 2.10 Fluorescein isothiocyanate‐dextran permeability assay

Two hours after LPS administration at last day during model embellishment, mice were given 750 mg/kg FITC‐dextran 4 kDa (FD‐4) by oral gavage and blood samples were collected 2 h after gavage. Then, the serum was separated and the concentration of FD‐4 in the serum was measured using a fluorescence spectrophotometer (Thermo Fisher Scientific, USA) at excitation and emission wavelengths of 490 and 520 nm, respectively.

### 2.11 Quantitative Real‐Time PCR

RNA was extracted from the cultured IEC‐6 cells and experimental mice using RNA‐easy™ Isolation Reagent (Vazyme, China) and subjected to reverse transcription (RT) with HiScript® III RT SuperMix for qPCR (+gDNA wiper) (Vazyme, China). The obtained cDNA was used for subsequent qRT‐PCR with Taq Pro Universal SYBR qPCR Master Mix (Vazyme, China). Relative quantities of mRNA and circRNAs were determined by the 2^−ΔΔ^CT method with glyceraldehyde‐3‐phosphate dehydrogenase (GAPDH) as the internal control. qRT‐PCR assay was conducted using the ABI 7500 system (ABI, USA) and the primer sequences of all targets were shown in Table S1.

### 2.12 Western blot

Whole‐cell lysates were prepared from IEC‐6 cells and intestinal tissues treated with various external stimulation to collect protein. Proteins were quantified using a bicinchoninic acid (BCA) protein assay kit (Vazyme, China), which were then subjected to sodium dodecyl sulfate polyacrylamide gel electrophoresis gel (SDS‐PAGE) and transferred to polyvinylidene fluoride (PVDF, Millipore, USA) membranes using an electroporation system. The membranes were blocked with 5% bovine serum albumin (Solarbio, China) in tris‐buffered saline with 0.1% Tween‐20 for 1 h at room temperature then incubated with specific primary antibodies against JNK (CST, USA), p‐JNK (CST, USA), claudin‐1 (ThermoFisher, USA), claudin‐3 (ThermoFisher, USA), claudin‐4 (ThermoFisher, USA), occludin (ThermoFisher, USA) and β‐actin (CST, USA) at 4°C overnight, followed by incubation in the presence of a horseradish peroxidase‐conjugated secondary antibody (CST, USA) for 2 h at room temperature. β‐actin was used as the internal reference for normalization. The protein bands were detected using an enhanced chemiluminescence kit (Millipore, USA) and visualized using a Bio‐Rad ChemiDoc XRS+ imaging system. The Image Lab and ImageJ software (Rockville, USA) was used for analysis.

### 2.13 Statistical analysis

All data are presented as the mean ± standard deviation (SD). SPSS version 22 software (IBM, USA) was used for statistical analyses, while the GraphPad Prism 9 software (La Jolla, USA) was used to acquire images. Survival analysis was performed using the Kaplan–Meier method. Differences among groups were compared using One‐way analysis of variance (anova) followed by Dunnett's test. *p* < 0.05 was the significance threshold.

## 3 RESULTS

### 3.1 Characteristics and distribution of CPJIP

**FIGURE 1:**
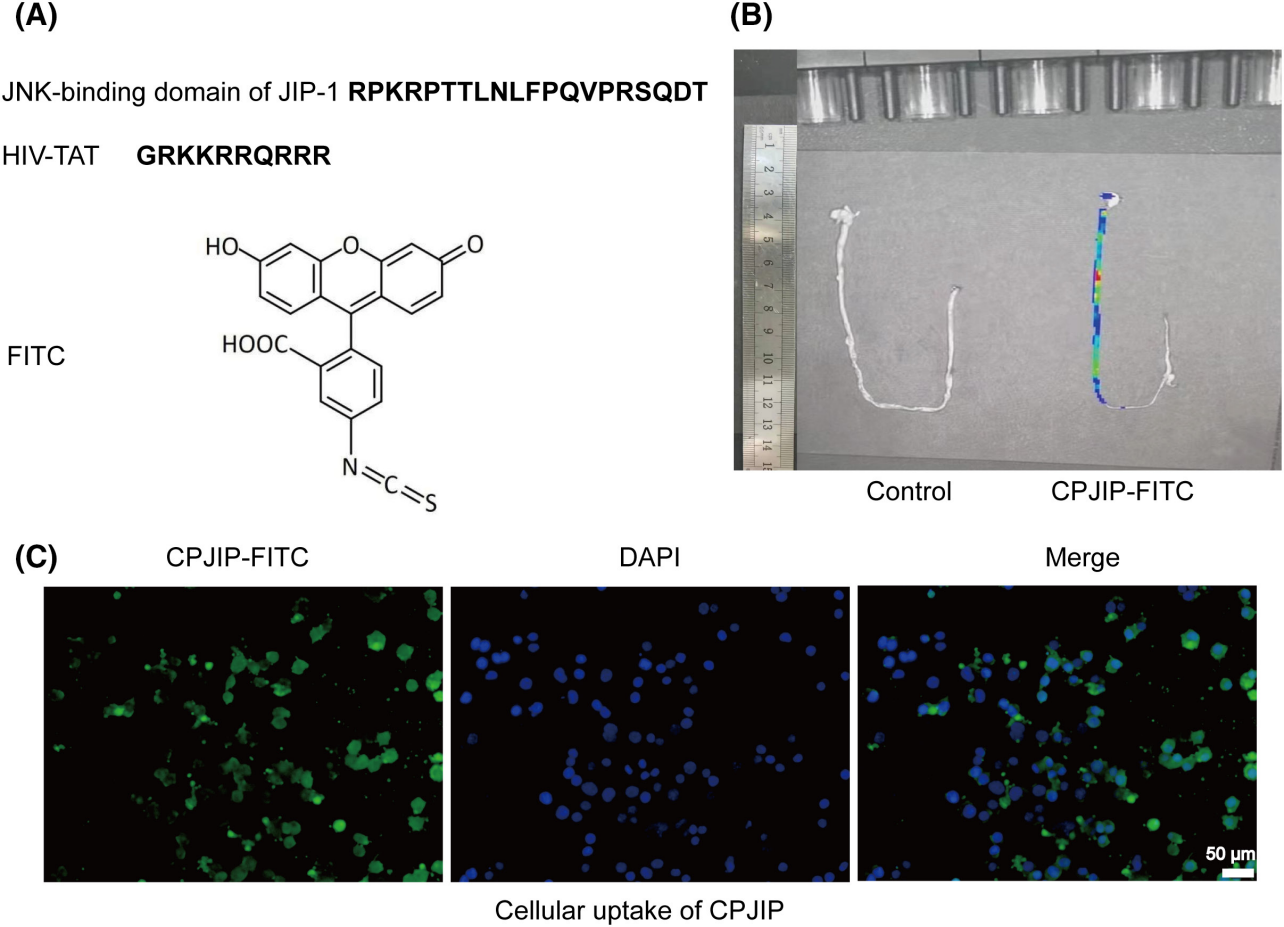
Characteristics and distribution of CPJIP. (A) Structure of CPJIP. (B) Fluorescence images of mice treated with FITC‐labelled CPJIP after 1 h of the oral test and control mice. (C) Cellular uptake of CPJIP in IEC6 cells. Cells were incubated with FITC‐labelled CPJIP (green) for 1 h and stained with DAPI (blue).

CPJIP is a synthesized peptide conjugated with FITC composed of 30 amino acids including a 20 amino acid listed from the JNK‐binding domain of JIP‐1 (RPKRPTTLNLFPQVPRSQDT) and a 10 amino acid derived from the HIV‐TAT sequence (GRKKRRQRRR), with a molecular weight of 3610 (Figure 1A). To assess in vivo distribution of CPJIP, mice were administrated with orally gavage of FITC‐labelled CPJIP for 1 h. As shown in Figure 1B, strong fluorescence signal was detected in intestinal tract of CPJIP‐FITC group, which implied a direct regulation of CPJIP on intestinal function in vivo. Since intestinal epithelial cells serve as an intestinal barrier component and play a critical role in NEC aetiology, we subsequently examined the cellular uptake efficiency of FITC‐CPJIP in IEC‐6 cells. Following a 1‐h incubation, a notable fluorescence expression of FITC‐CPJIP was observed in the cytoplasm and nucleus (Figure 1C). These results demonstrate that CPJIP is capable of entering into the target area with an advantage to act its biological function.

### 3.2 CPJIP inhibits JNK activation and ameliorates inflammation response as well as intestinal barrier in vitro

**FIGURE 2:**
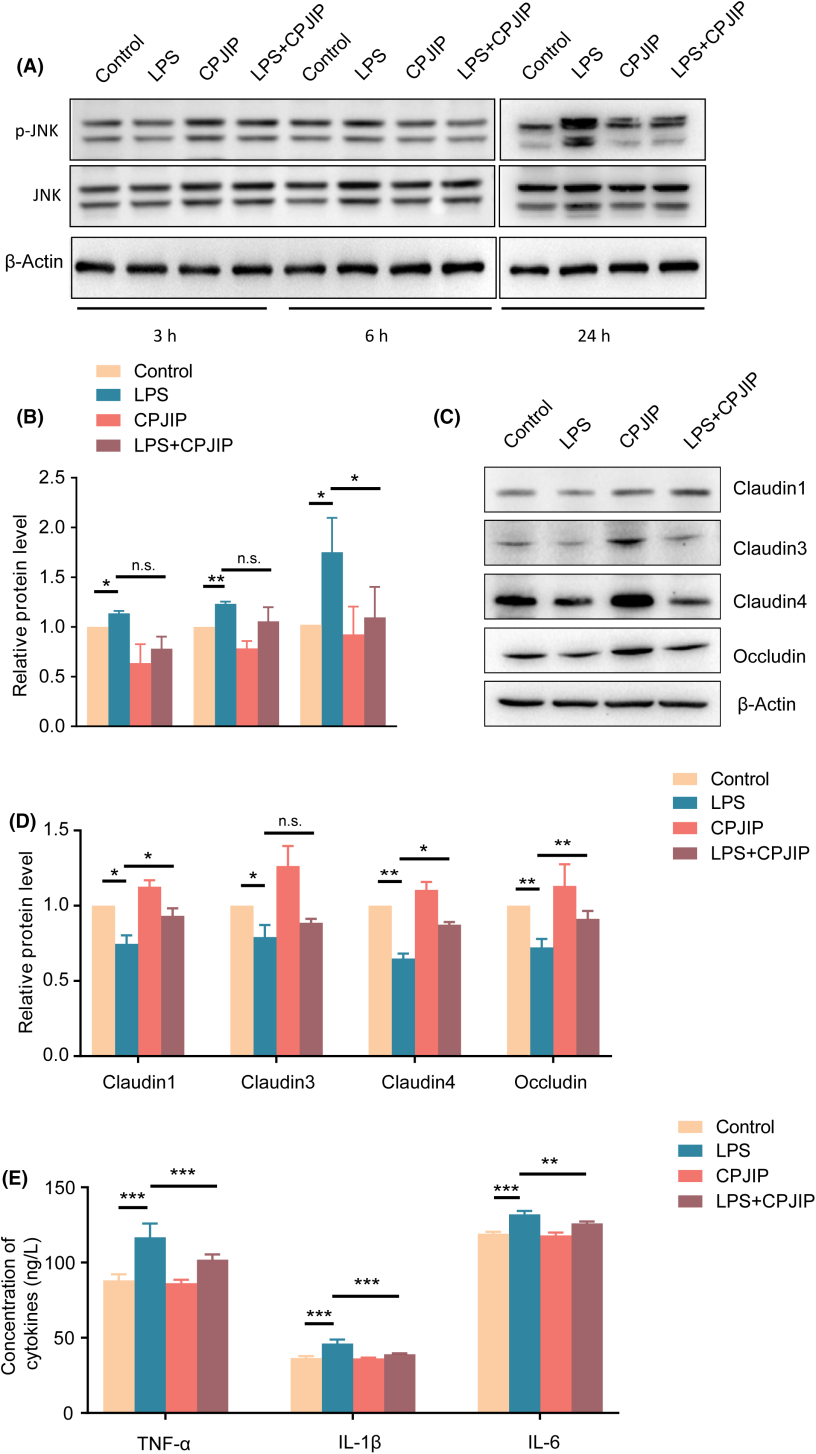
CPJIP inhibits JNK activation and ameliorates inflammation response as well as intestinal barrier in vitro. (A) Representative immunoblots images showing the protein expression of p‐JNK and JNK, (B) accompanied by quantification analysis of relative activation level of JNK. (C) Representative immunoblots images showing the protein expression of claudin‐1, claudin‐3, claudin‐4 and occludin in control, LPS, CPJIP and LPS + CPJIP groups, (D) along with quantification analysis; β‐Actin was used as a loading control. (E) The concentrations of inflammatory cytokines including TNF‐α, IL‐1β and IL‐6 in four groups were detected by ELISA kits. Data were shown as the mean ± SD. One‐way anova. n.s., not significant; *p < 0.05; **p < 0.01; ***p < 0.001.

It is well‐recognized that impaired intestinal barrier function, along with excessive inflammation, is widely observed in NEC. We first investigated whether CPJIP played regulatory roles in the activation of JNK pathway. The western blot results showed that LPS incubation for 3, 6 and 24 h all upregulated the phosphorylation of JNK (p‐JNK), however, CPJIP did not inhibit the increased p‐JNK level induced by LPS stimulation at 3 and 6 h (Figure 2A,B). Intriguingly, the p‐JNK expression was significantly decreased following CPJIP treatment for 24 h. Hence, we performed subsequent in vitro experiments employing an actuation duration for 24 h. To determine whether CPJIP improved intestinal epithelial barrier of NEC cell model, we then detected the protein expression of TJ components (claudin‐1, claudin‐3, claudin‐4 and occludin). The western blot results showed that the expression of all four TJ proteins were notably reduced after LPS treatment, which was significantly reversed except for claudin‐3 by administration of CPJIP (Figure 2C,D). In addition, we found that the production of the inflammatory factors TNF‐α, IL‐1β and IL‐6 was significantly increased, while CPJIP could attenuate the levels of three cytokines to inhibit inflammatory response. Collectively, all above data indicate that CPJIP suppresses JNK activation and restores intestinal barrier function by reducing inflammatory response in vitro.

### 3.3 CPJIP reduces the severity in a mouse NEC model

**FIGURE 3:**
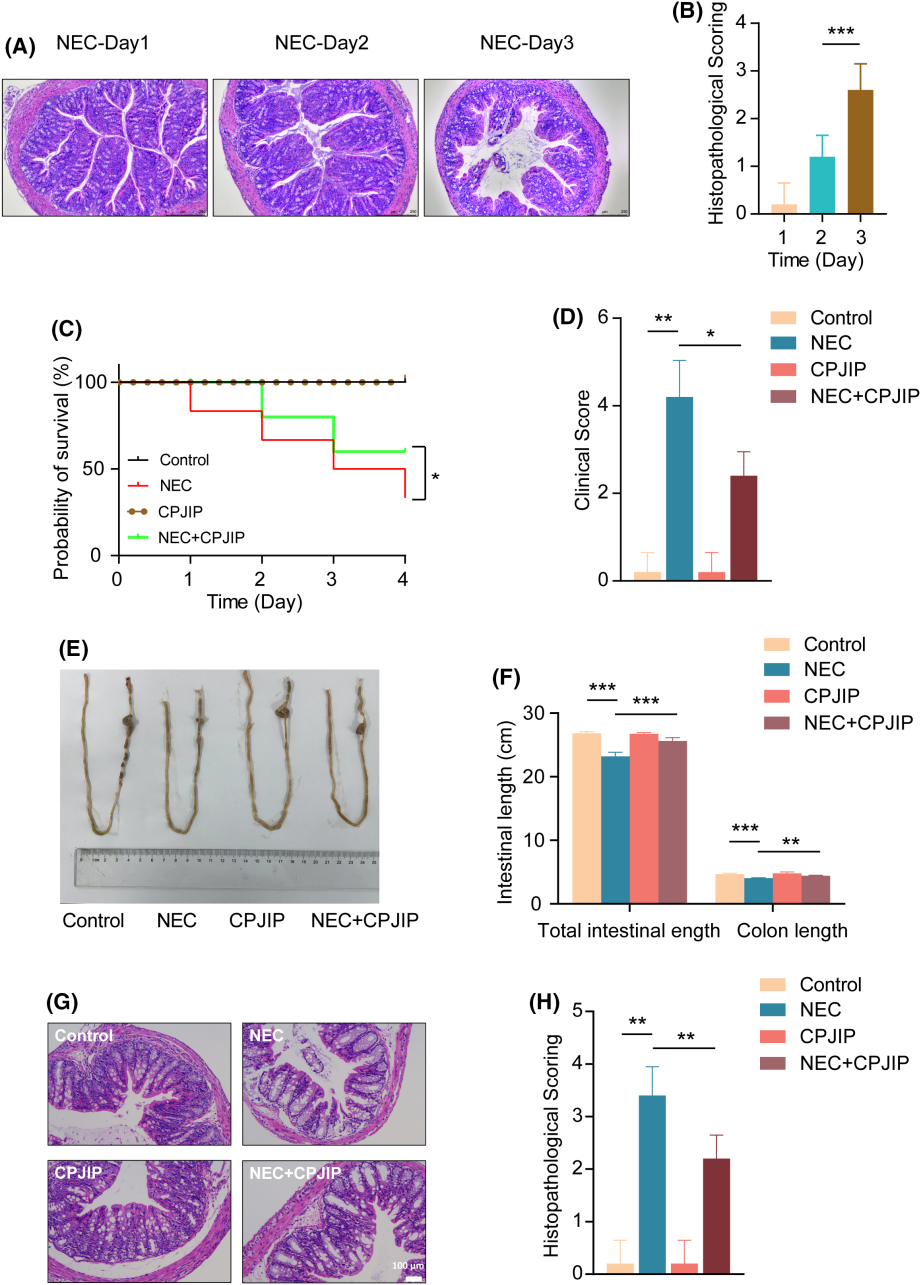
CPJIP lessened the severity of NEC mice. (A) Haematoxylin and eosin micrographs and (B) histopathological score of intestinal colon newborn rats subjected to procedure used to establish experimental NEC in gradually increasing days. (C) Survival curves fitted by a Kaplan–Meier plot showing the change tendency in control, NEC, CPJIP and NEC + CPJIP among which control and CPJIP group represented obvious overlap. (D) Clinical scores representing the baseline characteristics and clinical outcomes in control, NEC, CPJIP and NEC + CPJIP. (E) Representative photographs of the whole intestines of newborn mice followed by (F) quantification analysis of the total intestinal and colon length in four groups. (G) Representative photomicrographs and (H) histopathological score of colons derived from four individual groups showing the pathological changes and ameliorated manifestation. Data were shown as the mean ± SD. One‐way anova. *p < 0.05; **p < 0.01; ***p < 0.001.

To assess whether the experimental NEC was successfully established, haematoxylin and eosin staining was performed to intuitively observe pathological structural changes. As shown in Figure 3A,B, NEC mouse displayed progressive deterioration of intestinal tract characterized by swollen and broken villi, and disordered arrangement of lamina propria cells in days 1, 2 and 3. For another indication of NEC model, we found that the survival rate of NEC group was gradually decreased, but was significantly upregulated by administration of CPJIP in NEC mice (Figure 3C). Moreover, we performed clinical sickness scoring and found that NEC mice exhibited higher score, while CPJIP treatment could regain relatively low score indicating the alleviation of intestinal characters (Figure 3D). In addition to evaluating the overall features among four groups, we also measured the total intestinal and colon length. The results showed that both the total intestinal and colon lengths were lower in the NEC group than in the control and CPJIP groups, while that of NEC mice with CPJIP treatment was higher than in NA mice and control mice (Figure 3E,F). Haematoxylin and eosin staining data showed that the intestinal tissue of NEC mice exhibited an aberrant organization of epithelial cells, broken villi, partial necrosis, edema and slightly broken muscle and mucosal layer compared to control and CPJIP only groups. However, ameliorative microscopic intestinal mucosal injury and reduced pathological severity along with restored histopathological scores were observed in the NEC + CPJIP group (Figure 3G,H). In summary, these findings indicate that CPJIP improves intestinal development and ameliorates NEC severity in mouse model.

### 3.4 CPJIP improves intestinal barrier function by increasing TJ expression in NEC model

**FIGURE 4:**
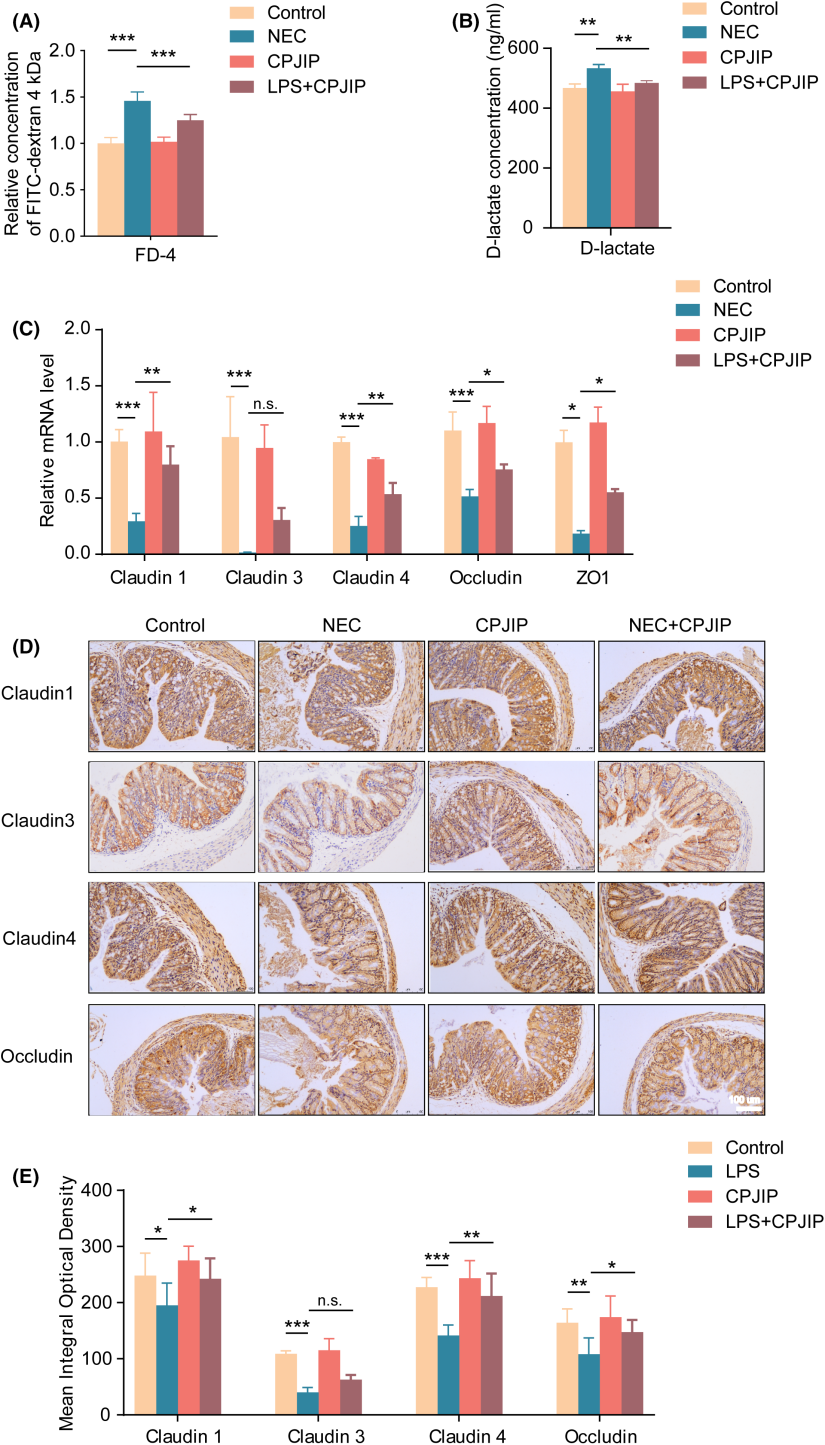
CPJIP improves intestinal barrier function by increasing tight junction (TJ) expression in NEC model. Detection of the levels of (A) FITC‐dextran 4 kDa and (B) D‐lactate in the serum of different groups reflecting the intestinal permeability. (C) qRT‐PCR analysis of TJ proteins including claudin‐1, claudin‐3, claudin‐4, occludin and ZO1. (D) Representative sections from colon tissue of control, NEC, CPJIP and NEC + CPJIP stained with anti‐claudin‐1, anti‐claudin‐3, anti‐claudin‐4 and anti‐occludin antibodies, along with (E) quantification analysis of mean integral optical densities. Scale bar: 100 μm. Data were shown as the mean ± SD. One‐way anova. n.s., not significant; *p < 0.05; **p < 0.01; ***p < 0.001.

This study indirectly evaluated intestinal barrier function through measuring intestinal permeability using serum FD‐4 and D‐lactate concentrations. Although D‐lactate is a metabolite of intestinal flora that cannot pass intact barriers, both FD‐4 and D‐lactate can enter the bloodstream through damaged or compromised intestines. The significant increase levels of serum FD‐4 (Figure 4A) and D‐lactate (Figure 4B) were observed, while these elevations were reversed using CPJIP. Meanwhile, we evaluated alterations of TJ proteins. The qRT‐PCR results showed that the mRNA expression of all TJ proteins, including claudin‐1, claudin‐3, claudin‐4, occludin and ZO1, was remarkably decreased in NEC mice. Unexpectedly, administration of CPJIP significantly restored the mRNA levels of TJ proteins except for claudin‐3 (Figure 4C). We also employed immunohistochemistry (IHC) to investigate the expression of TJ components (Figure 4D). Compared with the control group, the NEC mice exhibited notably reduced expression of claudin‐1, claudin‐3, claudin‐4 and occludin. Consistent with qRT‐PCR results, the mean integral optical densities of claudin‐1, claudin‐4 and occludin were elevated in NEC + CPJIP group compared to NEC group (Figure 4E). In addition, immunofluorescence of ZO‐1 and tricellulin was implemented to further support the alleviation of NEC symptoms following CPJIP administration (Figure S1). Taken together, these results suggested that CPJIP could enhance intestinal TJ function.

### 3.5 CPJIP attenuates intestinal inflammation and promotes intestinal cell growth partially via JNK pathway

**FIGURE 5:**
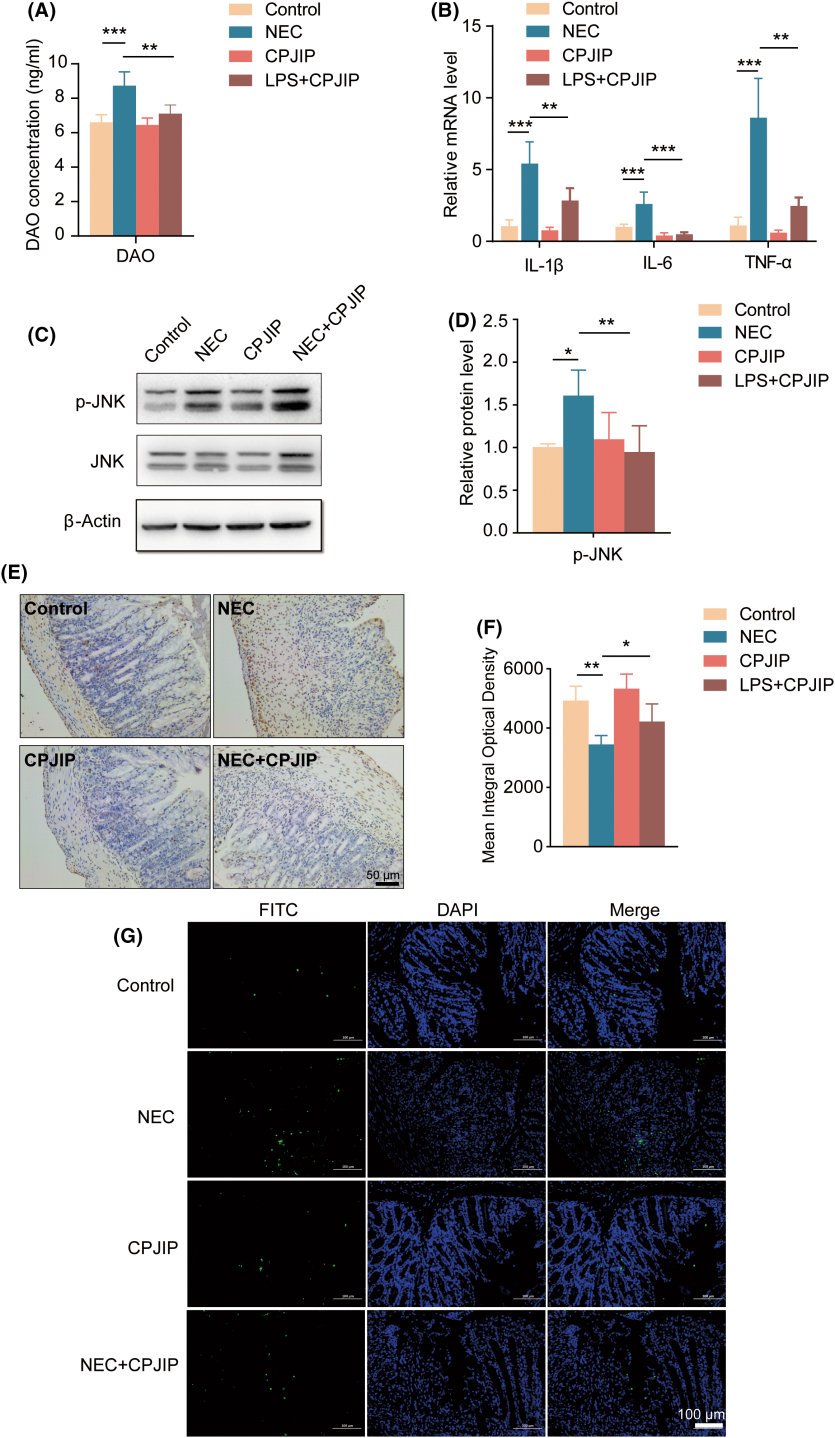
CPJIP attenuates intestinal inflammation and promotes intestinal cell growth partially via JNK pathway. (A) Measurement of serum levels of DAO using ELISA kit in different groups. (B) Detection of mRNA expression of inflammatory cytokines in intestinal colon tissues including IL‐1β, IL‐6 and TNF‐α by qRT‐PCR. (C) Representative immunoblots images showing the protein expression of p‐JNK, JNK and β‐actin, accompanied by (D) quantification analysis of relative activation level of JNK. (E) Representative sections from colon tissue of control, NEC, CPJIP and NEC + CPJIP stained with EdU incorporation, along with (F) quantification analysis of mean integral optical densities. (G) Representative image of TUNEL staining, green indicating TUNEL‐positive cells and blue indicating DAPI. Scale bar: 100 μm. Data were shown as the mean ± SD. One‐way anova. *p < 0.05; **p < 0.01; ***p < 0.001.

Normally, ciliated epithelial cells in the small intestinal mucosa express DAO, which is released into systemic circulation after massive epithelial damage. ELISA results showed a significant increase of DAO concentrations in NEC mice, but this augmentation was reversed by administering CPJIP (Figure 5A), implying that CPJIP has a protective effect against intestinal epithelial injuries. We then investigated the effect of CPJIP on cytokine production in NEC mouse intestinal tissue using qRT‐PCR and found that the production of the inflammatory factors IL‐1β, IL‐6 and TNF‐α was significantly increased in NEC than that in control group (Figure 5B). Importantly, when mice were treated with CPJIP during NEC modelling, the expression of these three cytokines was markedly decreased. To evaluate involvement of the JNK pathway in aetiology of NEC in vivo, Western blot was carried out (Figure 5C). The results showed that the phosphorylation of JNK (p‐JNK) was increased in NEC mice, while CPJIP inhibited the elevation of p‐JNK levels associated with NEC development (Figure 5D). Subsequently, we determined whether the aberrant cell viability of intestinal colon associated with pathogenesis of NEC was affected by CPJIP. The cell proliferation and apoptosis were measured using EdU staining and TUNEL assay. As shown in Figure 5E,F, the intestinal tissue of NEC mice exhibited significantly reduced cell proliferation compared to control mice, which was restored by administration of CPJIP under NEC condition. Moreover, TUNEL staining further revealed augmented cell apoptosis in NEC mice, whereas CPJIP elicited the opposite effect by inhibiting intestinal cell apoptosis (Figure 5G). Collectively, CPJIP improves intestinal inflammation‐induced injury and facilitates intestinal cell growth partially partly through JNK pathway.

## 4 DISCUSSION

NEC, a serious medical condition that primarily impacts newborns, exhibits a limited efficacy in treatment. Studies utilizing animal models demonstrate that enteral nutritional interventions have the potential to mitigate the occurrence, severity and manifestations of NEC. Furthermore, these interventions have been shown to ameliorate various pathological mechanisms associated with the progression of NEC, including intestinal inflammation and impairment of intestinal barrier function. In the present study, synthesized CPJIP was utilized in the context of the pathological progression of NEC, revealing its capacity to enhance the maintenance of the intestinal barrier by attenuating immune response and reducing the severity of NEC. It is noteworthy that the synthesized poly‐peptide CPJIP was an effective JNK inhibitor that was deeply investigated in the study of type 2 diabetes.^21^ Intriguingly, JNK signal pathway was demonstrated in a few articles to be implicated in the aetiology and pathogenesis of NEC. Moreover, JNK activity was associated with the regulation of inflammatory response that was also widely recognized as dominant contributor for NEC development. Hence, we here implemented experimental investigation on the regulatory function and underlying mechanism of CPJIP in the development of NEC. As to specificity, CPJIP is a derivative of the JNK binding domain of JNK‐interacting protein‐1 (JIP‐1), also referred to as islet‐brain‐1 (IB‐1), and has been first documented to act as a potent inhibitor of the JNK Pathway.^22^ Furthermore, our findings suggest that CPJIP may act as a potential therapeutic agent, in part through modulation of the JNK pathway. Therefore, it is hypothesized that the improved intestinal barrier function and reduced inflammation resulting from CPJIP administration may contribute to the amelioration of NEC severity.

The identification of a malfunctioning intestinal epithelial TJ barrier, coupled with aberrant expression of TJ proteins, has been recognized as a primary factor in the development of intestinal inflammation.^13^, ^23^, ^24^ Studies indicate that an increase in intestinal permeability due to the disruption of TJ barrier function, coupled with elevated levels of inflammatory cytokines, leukocyte infiltration and epithelial necrosis, may predispose premature infants to inflammatory diseases such as NEC and inflammatory bowel disease. Despite this risk, the valuable nutritional components present in maternal milk are beneficial for neonates.^25^, ^26^, ^27^, ^28^ In our study, we observed a consistent pattern of intestinal dysbiosis characterized by morphological damage, decreased expression of TJ proteins, heightened inflammatory response, impaired cell proliferation, and increased cell apoptosis in colon tissue. We hypothesize that bioactive peptides may serve a protective function in the gastrointestinal tract by resisting hydrolysis, promoting the maturation of the intestinal barrier and facilitating the absorption of peptides into systemic circulation and target tissues.^29^, ^30^ Peptides have been identified as potential anti‐oxidative agents that may mitigate the exacerbation of intestinal abnormalities,^19^, ^31^ implying the pivotal role of peptides in the pathogenesis of NEC. Previous studies have indicated that peptides with elevated isoelectric points, increased hydrophobicity or hydrophilicity, and reduced molecular weight exhibit a higher likelihood of traversing the CaCO_2_ cell monolayer via nonspecific fluid‐phase endocytosis, specific receptor‐mediated endocytosis, and carrier‐mediated transport.^32^, ^33^, ^34^ Our observations indicate that CPJIP can be transported into the intestinal local area in mice and the cytoplasmic domain in the CaCO_2_ cell line. Our current study demonstrates that CPJIP reduces inflammation in NEC and improves pathological features. Additionally, we found that CPJIP enhances overall survival and decreases the incidence of NEC. It is well‐established that defective intestinal barrier function contributes to NEC, and previous evidence suggests that there is no direct evidence of CPJIP's involvement in the re‐establishment of the underdeveloped physical barrier in NEC. Interestingly, CPJIP has been shown to enhance intestinal barrier function.

Studies have shown that inflammatory cytokines including TNF are increased in NEC, leading to activation of NF‐κB, JNK and p38 signalling pathways.^35^, ^36^ It is widely acknowledged that an impaired intestinal barrier function is associated with heightened JNK activity, resulting in dysbiosis and an exaggerated immune response, which can be ameliorated by inhibiting JNK.^37^ Moreover, the JNK pathway has the capacity to modulate the disassembly of TJ proteins. Activation of JNK can lead to the disruption of adherent junction protein interactions, phosphorylation of β‐catenin, and aberrant expression levels of TJ proteins, exacerbating the breakdown of epithelial junctions.^16^, ^38^ Concurrently, the administration of a JNK inhibitor enhanced the assembly of TJ proteins and improved the function of the intestinal barrier.^39^, ^40^ In addition to the regulatory effect of CPJIP on the enhancement of TJ protein levels, the permeable function related to TJ proteins also sparking the insight into the establishment procedure of NEC mice model about using hypertonic milk. It is well‐recognized that hypertonic microenvironment predisposes intestines of infant especially premature to mucosal injury, leading to progressive intestinal lesion. The procedure using hypertonic milk in mice for experimental NEC establishment was originally used by Jilling,^41^ which resulted in severe intestinal injury. Subsequent investigators combined hypertonic milk with combining with other manipulation including hypoxia and/or cold stimulation to further simulate pathological features characterized by disrupted intestinal structure and excessive inflammatory responses.

In the present study, a consistent increase in JNK activation and compromised intestinal barrier function in NEC was observed in both in vivo and in vitro. Additionally, the protective effect of CPJIP against NEC was shown to be associated with a reduction in JNK activation. However, the extent to which CPJIP improves disrupted intestinal function solely through the JNK pathway is not definitively supported by existing evidence, necessitating further experimental validation. These findings suggest that CPJIP may function as a potential JNK inhibitor in the context of NEC.

## 5 CONCLUSION

In summary, our study demonstrated that CPJIP effectively exerted anti‐inflammatory effects and improved intestinal barrier function by upregulating TJ protein expression in both in vivo and in vitro models. These effects were mediated, at least in part, by the inhibition of the JNK pathway and enhancement of intestinal cell viability, ultimately leading to a reduction in the severity of NEC. Our findings suggest that CPJIP may serve as a promising prophylactic treatment option for enhancing intestinal barrier function in infants at risk for NEC and preventing the onset of the disease. Additional evidence‐based support is required to elucidate the specific and exclusive mechanisms through which CPJIP acts as an effective regulator in protecting the intestinal system.

## AUTHOR CONTRIBUTIONS

**Le Zhang:** Conceptualization (lead); data curation (lead); funding acquisition (lead); project administration (lead); supervision (lead); writing – review and editing (lead). **Chaozhi Bu:** Formal analysis (lead); investigation (lead); methodology (lead); visualization (lead); writing – original draft (lead). **Mengyuan Hu:** Investigation (equal); methodology (equal); writing – original draft (equal). **Yinglin Su:** Investigation (equal); methodology (equal); writing – original draft (equal). **Fuqiang Yuan:** Formal analysis (supporting); visualization (equal). **Yiting Zhang:** Formal analysis (supporting); visualization (equal). **Jing Xia:** Conceptualization (equal); data curation (equal); supervision (equal); writing – review and editing (equal). **Zhenyu Jia:** Conceptualization (equal); data curation (equal); supervision (equal); writing – review and editing (equal).

## CONFLICT OF INTEREST STATEMENT

The authors declare no conflicts of interest.

## Supporting information


Figure S1.

Table S1.

## Data Availability

The datasets analyzed during the study are available from the corresponding author on reasonable request.

## References

[1] Stoll BJ , Hansen NI , Bell EF , et al. Trends in care practices, morbidity, and mortality of extremely preterm neonates, 1993–2012. JAMA. 2015;314:1039‐1051. 26348753 10.1001/jama.2015.10244 PMC4787615

[2] Neu J , Walker WA . Necrotizing enterocolitis. N Engl J Med. 2011;364:255‐264. 21247316 10.1056/NEJMra1005408 PMC3628622

[3] Neu J . Necrotizing enterocolitis: the future. Neonatology. 2020;117:240‐244. 32155645 10.1159/000506866

[4] Lin PW , Stoll BJ . Necrotising enterocolitis. Lancet (London, England). 2006;368:1271‐1283. 17027734 10.1016/S0140-6736(06)69525-1

[5] Turner JR . Intestinal mucosal barrier function in health and disease. Nat Rev Immunol. 2009;9:799‐809. 19855405 10.1038/nri2653

[6] Kaminsky LW , Al‐Sadi R , Ma TY . IL‐1β and the intestinal epithelial tight junction barrier. Front Immunol. 2021;12:767456. 34759934 10.3389/fimmu.2021.767456 PMC8574155

[7] Rawat M , Nighot M , al‐Sadi R , et al. IL1B increases intestinal tight junction permeability by up‐regulation of MIR200C‐3p, which degrades occludin mRNA. Gastroenterology. 2020;159:1375‐1389. 32569770 10.1053/j.gastro.2020.06.038 PMC11752806

[8] Al‐Sadi R , Ye D , Said HM , Ma TY . IL‐1beta‐induced increase in intestinal epithelial tight junction permeability is mediated by MEKK‐1 activation of canonical NF‐kappaB pathway. Am J Pathol. 2010;177:2310‐2322. 21048223 10.2353/ajpath.2010.100371 PMC2966790

[9] Moonwiriyakit A , Pathomthongtaweechai N , Steinhagen PR , Chantawichitwong P , Satianrapapong W , Pongkorpsakol P . Tight junctions: from molecules to gastrointestinal diseases. Tissue Barriers. 2023;11:2077620. 35621376 10.1080/21688370.2022.2077620 PMC10161963

[10] Hollander D . Intestinal permeability, leaky gut, and intestinal disorders. Curr Gastroenterol Rep. 1999;1:410‐416. 10980980 10.1007/s11894-999-0023-5

[11] Casirati A , Somaschini A , Perrone M , et al. Preterm birth and metabolic implications on later life: A narrative review focused on body composition. Front Nutr. 2022;9:978271. 36185669 10.3389/fnut.2022.978271 PMC9521164

[12] Bron PA , Kleerebezem M , Brummer RJ , et al. Can probiotics modulate human disease by impacting intestinal barrier function? Br J Nutr. 2017;117:93‐107. 28102115 10.1017/S0007114516004037 PMC5297585

[13] Moore SA , Nighot P , Reyes C , et al. Intestinal barrier dysfunction in human necrotizing enterocolitis. J Pediatr Surg. 2016;51:1907‐1913. 27720222 10.1016/j.jpedsurg.2016.09.011 PMC5245981

[14] Lessey LR , Robinson SC , Chaudhary R , Daniel JM . Adherens junction proteins on the move‐from the membrane to the nucleus in intestinal diseases. Front Cell Dev Biol. 2022;10:998373. 36274850 10.3389/fcell.2022.998373 PMC9581404

[15] Ornelas A , Dowdell AS , Lee JS , Colgan SP . Microbial metabolite regulation of epithelial cell‐cell interactions and barrier function. Cells. 2022;11:944. 10.3390/cells11060944 PMC8946845 35326394

[16] Deng J , Zeng L , Lai X , et al. Metformin protects against intestinal barrier dysfunction via AMPKα1‐dependent inhibition of JNK signalling activation. J Cell Mol Med. 2018;22:546‐557. 29148173 10.1111/jcmm.13342 PMC5742676

[17] Al‐Sadi R , Ye D , Boivin M , et al. Interleukin‐6 modulation of intestinal epithelial tight junction permeability is mediated by JNK pathway activation of claudin‐2 gene. PLoS One. 2014;9:e85345. 24662742 10.1371/journal.pone.0085345 PMC3963839

[18] Yan X , Cao Y , Chen W , et al. Peptide (tat(48‐60)) YVEEL protects against necrotizing enterocolitis through inhibition of toll‐like receptor 4‐mediated signaling in a phosphatidylinositol 3‐kinase/AKT dependent manner. Front Nutr. 2022;9:992145. 36299988 10.3389/fnut.2022.992145 PMC9590307

[19] Chen W , Chen Y , Qian Y , et al. The casein‐derived peptide YFYPEL alleviates intestinal epithelial cell dysfunction associated with NEC by regulating the PI3K/AKT signaling pathway. Food Funct. 2023;14:3769‐3778. 36995017 10.1039/d2fo02400d

[20] Agakidou E , Agakidis C , Kontou A , Chotas W , Sarafidis K . Antimicrobial peptides in early‐life host defense, perinatal infections, and necrotizing enterocolitis‐an update. J Clin Med. 2022;11:5074. 10.3390/jcm11175074 PMC9457252 36079001

[21] Kaneto H , Nakatani Y , Miyatsuka T , et al. Possible novel therapy for diabetes with cell‐permeable JNK‐inhibitory peptide. Nat Med. 2004;10:1128‐1132. 15448687 10.1038/nm1111

[22] Bonny C , Oberson A , Negri S , Sauser C , Schorderet DF . Cell‐permeable peptide inhibitors of JNK: novel blockers of beta‐cell death. Diabetes. 2001;50:77‐82. 11147798 10.2337/diabetes.50.1.77

[23] Zani A , Pierro A . Necrotizing enterocolitis: controversies and challenges. F1000Res. 2015;4:1373. 10.12688/f1000research.6888.1 PMC4753995 26918125

[24] de Lange IH , van Gorp C , Eeftinck Schattenkerk LD , van Gemert WG , Derikx JPM , Wolfs TGAM . Enteral feeding interventions in the prevention of necrotizing enterocolitis: a systematic review of experimental and clinical studies. Nutrients. 2021;13:1726. 10.3390/nu13051726 PMC8161173 34069699

[25] Quan R , Chen C , Yan W , Zhang Y , Zhao X , Fu Y . BAFF blockade attenuates inflammatory responses and intestinal barrier dysfunction in a murine endotoxemia model. Front Immunol. 2020;11:570920. 33324396 10.3389/fimmu.2020.570920 PMC7725703

[26] Gunasekaran A , Eckert J , Burge K , et al. Hyaluronan 35 kDa enhances epithelial barrier function and protects against the development of murine necrotizing enterocolitis. Pediatr Res. 2020;87:1177‐1184. 31499514 10.1038/s41390-019-0563-9 PMC7061074

[27] Fan H , Chen Z , Lin R , et al. Bacteroides fragilis strain ZY‐312 defense against *Cronobacter sakazakii*‐induced necrotizing enterocolitis in vitro and in a neonatal rat model. mSystems. 2019;4:e00305. 31387931 10.1128/mSystems.00305-19 PMC6687943

[28] Bein A , Eventov‐Friedman S , Arbell D , Schwartz B . Intestinal tight junctions are severely altered in NEC preterm neonates. Pediatr Neonatol. 2018;59:464‐473. 29276042 10.1016/j.pedneo.2017.11.018

[29] Wada Y , Lönnerdal B . Bioactive peptides derived from human milk proteins—mechanisms of action. J Nutr Biochem. 2014;25:503‐514. 24411973 10.1016/j.jnutbio.2013.10.012

[30] Wada Y , Lönnerdal B . Bioactive peptides derived from human milk proteins: an update. Curr Opin Clin Nutr Metab Care. 2020;23:217‐222. 32068546 10.1097/MCO.0000000000000642

[31] Chen Y , Fantuzzi G , Schoeny M , Meier P , Patel AL . High‐dose human milk feedings decrease oxidative stress in premature infant. JPEN J Parenter Enteral Nutr. 2019;43:126‐132. 29761879 10.1002/jpen.1178

[32] Wang B , Li B . Charge and hydrophobicity of casein peptides influence transepithelial transport and bioavailability. Food Chem. 2018;245:646‐652. 29287421 10.1016/j.foodchem.2017.09.032

[33] Pardridge WM , Boado RJ . Enhanced cellular uptake of biotinylated antisense oligonucleotide or peptide mediated by avidin, a cationic protein. FEBS Lett. 1991;288:30‐32. 1879560 10.1016/0014-5793(91)80996-g

[34] Liang N , Kim BJ , Dallas DC . Bioavailability of peptides derived from the in vitro digestion of human milk assessed by Caco‐2 cell monolayers. J Agric Food Chem. 2022;70:7077‐7084. 35608530 10.1021/acs.jafc.2c01246

[35] Jacobs AT , Ignarro LJ . Nuclear factor‐kappa B and mitogen‐activated protein kinases mediate nitric oxide‐enhanced transcriptional expression of interferon‐beta. J Biol Chem. 2003;278:8018‐8027. 12500976 10.1074/jbc.M211642200

[36] Blaser H , Dostert C , Mak TW , Brenner D . TNF and ROS crosstalk in inflammation. Trends Cell Biol. 2016;26:249‐261. 26791157 10.1016/j.tcb.2015.12.002

[37] Zhou J , Boutros M . JNK‐dependent intestinal barrier failure disrupts host‐microbe homeostasis during tumorigenesis. Proc Natl Acad Sci USA. 2020;117:9401‐9412. 32277031 10.1073/pnas.1913976117 PMC7196803

[38] Lee MH , Koria P , Qu J , Andreadis ST . JNK phosphorylates beta‐catenin and regulates adherens junctions. FASEB J. 2009;23:3874‐3883. 19667122 10.1096/fj.08-117804 PMC2774999

[39] Samak G , Chaudhry KK , Gangwar R , Narayanan D , Jaggar JH , Rao RK . Calcium/Ask1/MKK7/JNK2/c‐Src signalling cascade mediates disruption of intestinal epithelial tight junctions by dextran sulfate sodium. Biochem J. 2015;465:503‐515. 25377781 10.1042/BJ20140450 PMC4385020

[40] Naydenov NG , Hopkins AM , Ivanov AI . c‐Jun N‐terminal kinase mediates disassembly of apical junctions in model intestinal epithelia. Cell Cycle. 2009;8:2110‐2121. 19502798 10.4161/cc.8.13.8928

[41] Jilling T , Simon D , Lu J , et al. The roles of bacteria and TLR4 in rat and murine models of necrotizing enterocolitis. J Immunol. 2006;177:3273‐3282. 16920968 10.4049/jimmunol.177.5.3273 PMC2697969

