# Supplementary figures and images for: Cell‐permeable JNK‐inhibitory peptide regulates intestinal barrier function and inflammation to ameliorate necrotizing enterocolitis
Source: J Cell Mol Med. 2024 Jul 19;28(14):e18534. doi: 10.1111/jcmm.18534 (PMC11258882; doi:10.1111/jcmm.18534)

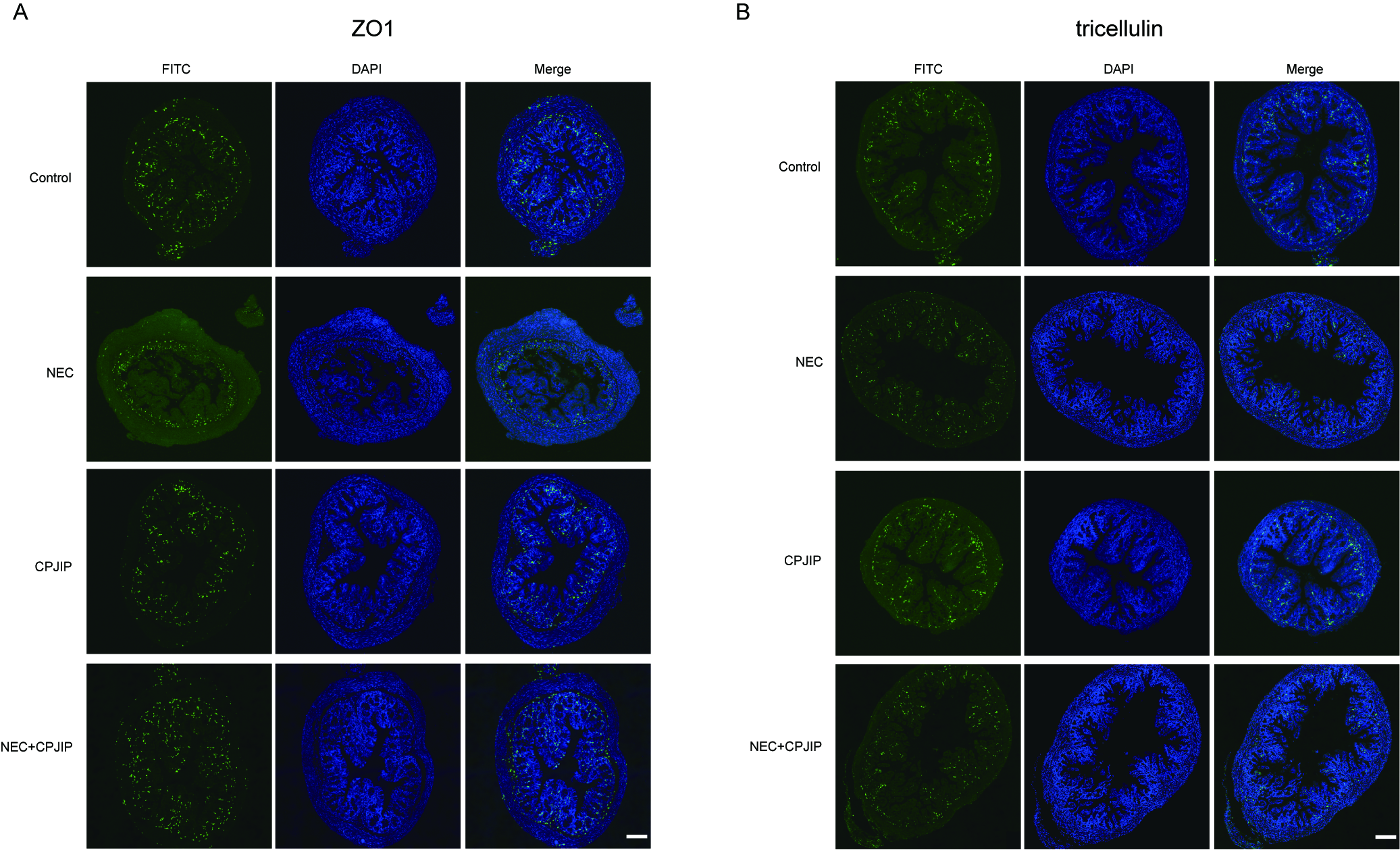

Supplement: Supplementary file 1 — Figure S1. [file JCMM-28-e18534-s001.tif]
